# Developing and Testing the Effectiveness of a Novel Health Qigong for Frail Elders in Hong Kong: A Preliminary Study

**DOI:** 10.1155/2013/827392

**Published:** 2013-09-10

**Authors:** Hector W. H. Tsang, Janet L. C. Lee, Doreen W. H. Au, Karen K. W. Wong, K. W. Lai

**Affiliations:** ^1^Neuropsychiatric Rehabilitation Laboratory, Department of Rehabilitation Sciences, The Hong Kong Polytechnic University, Hung Hom, Hong Kong; ^2^Yan Chai Hospital Social Services Department, Yan Chai Hospital, Tsuen Wan, Hong Kong

## Abstract

Eight-Section Brocades and Yijin Jing consist of some routine movements that are too difficult for frail elders. A novel health qigong protocol was developed and its effectiveness for frail elders was examined using a randomized clinical trial (RCT). An expert panel performed functional anatomy analysis and safety field test prior to the RCT. The experimental group (*n* = 61, 83 ± 6 yr) was given a 12-week qigong exercise program, while the comparison group (*n* = 55, 84 ± 6 yr) participated in a newspaper reading program with the same duration and frequency. Pre-, mid-, post-, and follow-up assessments were conducted. At 12 weeks, the qigong group had significant improvements in thinking operations (*F* = 4.05, *P* = .02) and significant reduction of resting heart rate (*F* = 3.14, *P* = .045) as compared to the newspaper reading group. A trend of improvements in grip strength and a decreasing trend of depression levels were observed among the qigong group. Significant perceived improvements in physical health (*F* = 13.01, *P* = .001), activities of daily living (*F* = 5.32, *P* = .03), and overall health status (*F* = 15.26, *P* = .0001) were found. There are improvements in some aspects of psychosocial, cognitive, physical, and physiological domains. Clinical applications and possibilities for further research are discussed.

## 1. Introduction

The aging population around the globe will soar from 37.3 years in 2000 to 45.5 years in 2050 [1] which will be accompanied by various levels of frailty and eventually increase utilization of health care services [2, 3]. Frail elders are at higher risk of physical and cognitive decline, disability, and finally death [4, 5]. Physical therapy and cognitive training programs are mainstream interventions to prevention of functional decline in frail elders [6–8]. Previous studies however focused only on either the physical or cognitive aspect of the frail elders. To date, no studies have been found to address both aspects at the same time with one treatment program. Studies have shown that compliance was low among physically frail elders on convention programs [6]. Because of these limitations, there is an enormous need to explore alternative and complementary ways of reducing frailty and the related disabilities which would then minimize burden on the health care system. 

Health qigong, a form of mind-body intervention, is demonstrated to improve physical and psychosocial functions. Eight-Section Brocades is the most widely practiced qigong protocol among older adults which may help elders adapt stress, improve neurohormonal regulation, and strengthen their cardiovascular functions [9]. Similarly, Yijin Jing has received consistent and compelling scientific evidence of health benefits on homeostasis of internal organs and enhancement of physiologic capacity of individuals [9]. Clinical experiences suggest however that both Eight-Section Brocades and Yijin Jing have limitations as these protocols have some routine movements that are too difficult for the frail elderly. To address this concern, we developed a novel health qigong protocol, the “Yan Chai Yi Jin Ten-Section Brocades,” putting together the easier and more suitable routines of these two well-known qigong protocols. 

This paper reported the development of this new qigong protocol and adopted a randomized clinical trial (RCT) to examine its effectiveness for frail elders in the aspects of psychosocial, cognitive, physical, and physiological functioning. 

## 2. Methods

### 2.1. Development of the Yan Chai Yi Jin Ten-Section Brocades

A core group involving the first author, a TCM practitioner, and an occupational therapist (OT) was formed to develop initial protocols for both sitting and standing positions. Ten easier routines suitable for frail elders covering movement of major joints all over the body were selected from the Eight-Section Brocades and Yijin Jing. The ten sequential movements were from upper limbs to lower limbs with five easier routines selected from each of the two established protocols (Table 1). An expert panel consisting of six researchers and practitioners with diverse expertise was then formed. The panel consisted of a qigong researcher, two OTs, a social worker, a physiotherapist, a psychologist, and a TCM practitioner. The members were invited to assess the therapeutic effects of the new protocols from various health and safety aspects [10]. All panel members evaluated 17 potential health effect statements for both positions using content validation ratios (CVRs). The CVRs of 0.75 or above were indicated as significant agreement among six experts. Fourteen out of 17 statements received CVR ranging from 0.83 to 1.00 which suggested that the new protocols could facilitate relaxation, relieve unpleasant feelings, arouse cultural interests, enforce “deep and slow” breathing, enhance coordination between respiration and movements, promote good health through reciprocal movements, promote physical and psychological health, and prevent potential harms (Table 2). Two experts in OT and physiotherapy were selected to perform a functional anatomy analysis for both positions. With the concordance rate ranging from 85.05% to 92.13%, both experts confirmed that all major joints including neck, shoulder, elbows, fingers, wrist, spine, hip, and knee were involved to provide health benefits for frail elders. A field test was arranged to assess the physiological responses of the new protocols against the standard safety criteria [11]. A total of 11 elders were referred by the residential homes to take part in the field test for both standing and sitting positions at separate sessions. Both sessions were led by a certified health qigong instructor and each session lasted for an hour. The referred elders were females aged between 77 to 95 years (mean = 86 years, SD = 5 years). Physiological responses were assessed individually before and after the practice. One elder from the sitting group was excluded from the field test because hypertensive response (SBP > 200) was detected prior to the practice. A compliance rate to safe practice was 100% for six criteria which included no chest pain nor dizziness, no signs of insufficient blood circulation, oxyhemoglobin saturation by pulse oximetry (SpO_2_) above 90%, no palpitation together with irregular heart rate pattern, rise in heart rate within 70% of heart rate reserve, and rating of perceived exertion below level 7 on the 10-point Borg Scale. One elder from each of the standing group and sitting group showed hypertensive response after the practice. No other adverse events or maladaptive responses were observed during the field test. It was therefore concluded that the developed health qigong protocol “Yan Chai Yi Jin Ten-Section Brocades” was safe and suitable for frail elders including those who were wheelchair bound.

### 2.2. Study Design

An RCT was conducted to examine the effectiveness of the Yan Chai Yi Jin Ten-Section Brocades for the frail elders in terms of its beneficial effects on the psychosocial, cognitive, and physical functioning.

### 2.3. Sample Size Justification

The psychosocial functioning outcome on Geriatric Depression Scale (GDS) was used for sample size estimation. Using Cohen's method [12] effect sizes of these outcome measures are found to range from .25 to .29. The effect size of .25 was used for the calculation, as it is a commonly accepted principle to adopt smaller effect size for sample size estimation. With the help of power analysis and sample size (PASS), assuming power =  .80, type I error = .05, and a 10% drop-out rate, at least 70 participants for each group making a total of 140 participants should be recruited from the Elderly Division of YCHSSD. A subsample of participants would receive physiological measures on their stress responses and cardiopulmonary functions. According to previous studies [13, 14] and practical concerns, it is estimated that approximately 40 to 50 participants should be engaged in more comprehensive physiological measures using polygraph, ultrasonoscope, and microspirometer.

### 2.4. Participants and Settings

A total of 182 elders aged 60 and over were recruited from the Elderly Service Unit from the Yan Chai Hospital Social Services Department in Hong Kong. The selection criteria included those who (1) aged 60 and over and (2) obtained a score of 8 or higher in 62-item frailty index. A total of 134 eligible participants (36 males, 98 females) from an original sample of 182 participants were eventually included in this study from March 2012 to July 2012. They were then randomly assigned to either the intervention group or the control group. 18 out of 134 eligible participants (13%) dropped out from the study because of returning home, hospitalization, or moving to other centers. A total of 116 participants were eventually involved in the study.  Figure 2 reports the CONSORT diagram for the recruitment and randomization process.

### 2.5. Measurements

#### 2.5.1. Psychosocial Functioning

The 15-item Geriatric Depression Scale (GDS) [15] was used to assess depressed mood of participants. A score of 8 or above indicated presentation of clinical depression symptoms. This scale was reported to have good reliability in a validation study conducted in Hong Kong [16]. 

The 21-item Perceived Benefit Questionnaire (PBQ) [17] was adopted to measure the perceived improvement in physical health, activities of daily living, psychological health, social relationship, and health in general of participants for the qigong practice group. The coefficient alpha and test retest reliability for this questionnaire were .88 and .91, respectively.

#### 2.5.2. Cognitive Functioning

Lowenstein Occupational Therapy Cognitive Assessment-Geriatric (LOTCA-G) [18] was used to assess cognitive functioning of the participants. It consisted of 23 subsets on orientation, visual and spatial perception, praxis, visuomotor organization, and so forth, of the elders. LOTCA-G was reported to be a sound cognitive assessment tool among elders with good concurrent validity [19].

#### 2.5.3. Physical Functioning

Handgrip strength provided an objective assessment of the subjects' general level of muscle strength [20]. The Jamar hydraulic dynamometer (Bolingbrook, IL) was used to test the maximum handgrip strength of both hands of each subject [21]. 

The Timed Up and Go test was used as a sensitive and valid measure for identifying older adults at risk of falls [22]. Three trials were timed for each subject and the mean value is calculated for comparison. 

#### 2.5.4. Physiological Parameters

Heart rate and systolic and diastolic blood pressure were measured by the OMRON electronics blood pressure monitor (model: BP724). Pulmonary function was assessed by lung capacity and circulation ability by a microspirometer. Lung capacity was measured by the maximum forced vital capacity (FVC). Circulation ability was evaluated by the forced expiratory volume in one second (FEV1). 

### 2.6. Intervention Program

A 12-week intervention program was given to the participants at the corresponding day centers and residential care homes. Twenty-four intervention sessions were offered to the participants with two supervised 60-minute sessions per week. Participants assigned to the intervention group were provided with the Yan Chai Yi Jin Ten-Section Brocades in group format led by five qualified qigong instructors. Participants in the comparison group were assigned to a newspaper reading activity led by staff from elderly residential homes and day care center that had experience in leading rehabilitation activities.

#### 2.6.1. Qigong Exercise Group

**Figure 1:**
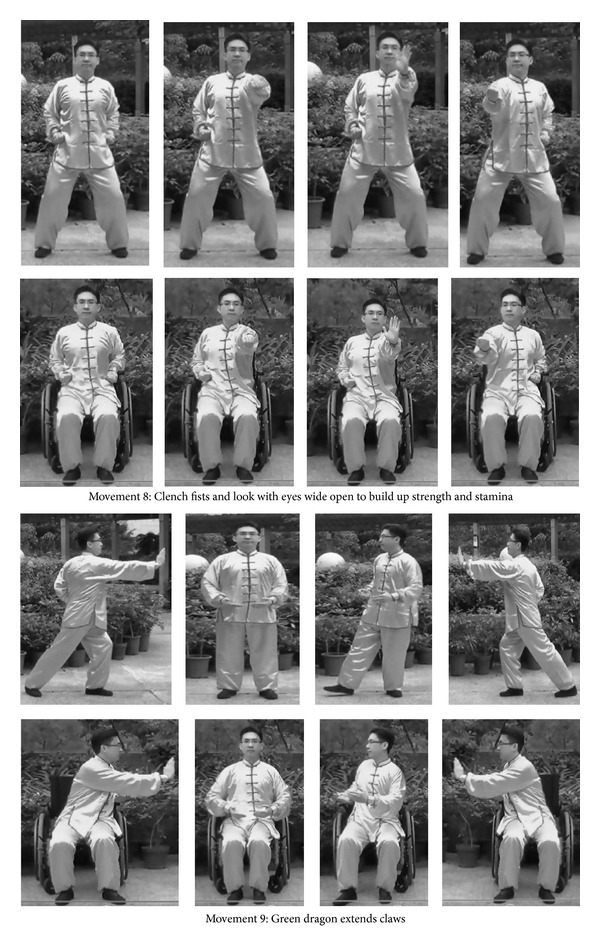
Yan Chai Yi Jin Ten-Section Brocade with selected illustrations for standing and sitting positions.

**Figure 2:**
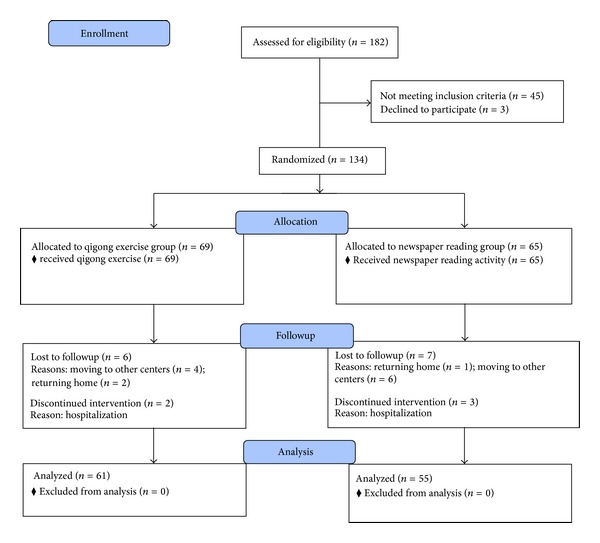
Consort flow diagram.

The Yan Chai Yi Jin Ten-Section Brocades protocol consisted of ten sequential forms of movements which was either practiced in both standing and sitting styles depending on the participants' abilities (see Figure 1). A complete cycle of the Yan Chai Yi Jin Ten-Section Brocades took 10 to 15 minutes. Each training session lasted for 60 minutes. Participants were led by certified qigong instructor to practice each of the movements of the qigong protocol 2-3 times with guided practice on mindfulness and rhythmic breathing at the beginning and short breaks between successive cycles. Participants were encouraged to practice qigong daily after the intervention program throughout the project period.

#### 2.6.2. Newspaper Reading Group

Each session lasted for 60 minutes. The instructor read aloud the newspaper articles chosen from the news headlines during the week. The participants were asked to answer brief questions about the article and express their views. The newspaper reading and discussion activity was chosen as a comparison group activity because it was a basic rehabilitation activity that was often provided in geriatric settings and was able to neutralize the attention given by therapist compared with the experimental group. It was considered by international experts to be a good comparison activity in previous studies [23, 24]. The duration and frequency were identical to the intervention group. 

### 2.7. Data Collection

Informed written consent was obtained from participants following policies of the institutional review board of the facilities. All participants completed the psychosocial, cognitive, and physical functioning assessments, with 57 (49.1%) participants taking part in the clinical assessments of physiological functioning (i.e., pulmonary function). All assessors were blinded as to the group assignment of the participants. The psychosocial functioning measures were collected by trained independent assessors via face-to-face interview before commencement of the intervention program (preassessment), after the 6th week of the program (mid-assessment), immediately after the end of the program (postassessment), and eight weeks after the completion of the program (follow-up assessment). The physiological measures were obtained before the commencement (preassessment) and immediately after the end of the program (postassessment). The assessment procedures were approved by the ethics committee of the authors' affiliated university and the institutional review board of the facilities. 

### 2.8. Data Analysis

Predictive Analytics Software (PASW) 20 was used for data analysis. The outcome measures of this study included psychosocial, cognitive, physical, and physiological functioning. The intervention effects were examined by group × time interaction effects with repeated measures ANOVA/ANCOVAs followed by post hoc analyses where appropriate. The baseline scores were treated as covariates if significant group differences were detected by independent *t*-tests. Partial eta-squared (*η*_*p*_^2^) was adopted for the estimation of unbiased effect size of the intervention [25]. A two-stage analysis was adopted to compare differences between the two groups in terms of the acute intervention effects (from baseline to postassessment) and the maintenance effects (from post- to follow-up assessment). The outcome measures with significant acute intervention effects at the postassessment were included for the examination of the maintenance effects. All analyses followed the principle of “intent-to-treat” analysis. Missing data in mid- and postassessment were replaced using the “Last-Observation-Carried-Forward (LOCF)” method. Significant levels were set at *P* < .05 for all analyses. Using the median score obtained in a large-scale local study in 2,032 elders aged 70 or above [26], only participants with a frailty index score of eight or above were included in the analysis.

## 3. Results

### 3.1. Demographics Characteristics

The demographic information of the participants is summarized in Table 3. Comparison of the two groups did not reveal significant differences in demographic characteristics (*P*s = .052 to .93). The mean age of experimental group participants (*N* = 61) was 83.3 (SD = 6.30), and that of the comparison group participants (*N* = 55) was 84.9 (SD = 6.03). The mean rating for the mini-mental state examination (MMSE) score of experimental group participants was 23.69 (SD = 3.52) and that of the comparison group participants was 23.58 (SD = 3.61), which suggested that the participants ranged from mild cognitive impairment to normal cognitive functioning subject to their educational levels [27]. The mean rating for the Clifton assessment procedures for the elderly (CAPE) score of experimental group was 9.21 (SD = 2.08) and that of the comparison group was 9 (SD = 1.98), meaning no mental impairment and no significant behavioral disability. Sixty-one (52.6%) experimental group participants and fifty-five (47.4%) comparison group participants scored eight or above in the frailty index, meaning the frailty level was higher than 50% of the elderly population. There were no reports of adverse events on both groups of participants during the implementation of interventions and assessments throughout the study.

### 3.2. Acute Intervention and Maintenance Effects on Psychosocial Measures

Repeated measures ANOVA within the experimental group in the acute intervention stage (Table 4) revealed significant across time effects on self-perceived benefits on physical health [*F*(1,54) = 13.01, *P* = .001, *η*_*p*_^2^ = .19], activities of daily living [*F*(1,43) = 5.32, *P* = .03, *η*_*p*_^2^ = .11] and overall health status [*F*(1,57) = 15.26, *P* = .0001, *η*_*p*_^2^ = .21]. From mid-assessment to postassessment, post hoc analyses found that the experimental participants had significantly higher level of self-perceived benefits on physical health (17.09 versus 18.05, resp.), activities of daily living (12.98 versus 13.48, resp.), and overall health status (6.95 versus 7.43, resp.). The self-perceived benefits on physical health were increased by 5.7%, activities of daily living was increased by 3.9%, and overall health status was increased by 6.9%. No significant across time effects on GDS [*F*(2,228) = 1.16, *P* = .32, *η*_*p*_^2^ = .01], self-perceived benefits on psychological [*F*(1,57) = .05, *P* = .82, *η*_*p*_^2^ = .00], and social relationship [*F*(1,57) = .32, *P* = .58, *η*_*p*_^2^ = .01] were found. A decreasing trend of levels of GDS was however observed among the experimental participants from mid-assessment to postassessment (from 4.61 to 4.31), whereas an increasing trend was observed in the comparison group participants (from 4.24 to 4.76). As to the maintenance effect, there were no significant across time effects on the perceived benefits on physical health [*F*(1,53) = 2.82, *P* = .10, *η*_*p*_^2^ = .05], activities of daily living [*F*(1,45) = .15, *P* = .70, *η*_*p*_^2^ = .00], and overall health status [*F*(1,59) = .67, *P* = .42, *η*_*p*_^2^ = .01].

### 3.3. Acute Intervention and Maintenance Effects on Cognitive Measures

A significant group by time interaction effect was indicated for thinking operations [*F*(2,228) = 4.05, *P* = .02, *η*_*p*_^2^ = .03], with an average increase of 8.6% (i.e., from 4.41 in preassessment to 4.79 in postassessment) observed among the experimental participants compared to an average decrease of 7.5% (i.e., from 4.56 in preassessment to 4.22 in postassessment) observed among the comparison participants (Table 5). Post hoc analyses found that experimental participants had significantly better thinking operations (*F* = 7.87, *P* < 0.025 with Bonferroni adjustment, *η*_*p*_^2^ = .07) in the mid-assessment. Repeated measures ANOVAs revealed no overall significant group by time interaction effects on measurements of orientation, perception, praxis, visuomotor organization, memory and attention (*P*s > .05). However, from preassessment to postassessment, experimental participants were remarkably improved in perception than in comparison participants. The experimental participants had an average increase of 1.7% in perception domain, while comparison participants had an average increase of 0.5% only. As the maintenance effect, repeated measures ANOVAs revealed a nonsignificant effect on the thinking operations [*F*(1,113) = 3.57, *P* = .06, *η*_*p*_^2^ = .03].

### 3.4. Acute Intervention and Maintenance Effects on Physical and Physiological Measures

A comparison of intervention effects between groups in the acute intervention stage showed significant group by time interaction effects on resting heart rate (RHR) [*F*(2,228) = 3.14, *P* = .045, *η*_*p*_^2^ = .03]. Experimental participants had an average reduction of 2.9% in RHR after 12-week qigong practice (i.e., from 76.39 bpm in preassessment to 73.51 bpm in postassessment), while RHR of comparison group remains unchanged after the 12-week intervention period (Table 6). Although not statistically significant (*P* = 0.58), there was a trend of improvement in right handgrip strength among the experimental group participants by 1.43% on average (i.e., from 16.79 kg in preassessment to 17.03 kg in postassessment). For the comparison group, the participants had 2.47% of decrease on average from 15.37 kg in preassessment to 15 kg in postassessment (i.e., from 15.37 kg in preassessment to 15 kg in postassessment). Repeated measures ANOVAs did not reveal significant maintenance effect on the resting heart rate [*F*(1,114) = 0.22, *P* = .64, *η*_*p*_^2^ = .22].

## 4. Discussion

The present study demonstrated the impact of the novel health qigong on psychosocial, cognitive, physical, and physiological domains in a sample of frail elders in Hong Kong. Positive intervention effects were found in some aspects of psychosocial, cognitive, physical, and physiological domains which provided preliminary support to its potential benefits as a therapeutic activity for frail elders. 

Studies on cognitive benefits of mind-body exercises are limited. Most studies reported only self-perceived benefits of qigong on cognitive functioning [28–30]. While earlier studies showed that Tai Chi and Shaolin Dan Tian Breathing reduced cognitive impairment and induce attentive state of mind [31, 32], the present study went further to explore the impact of qigong exercises on cognitive functioning using objective assessment. We showed that after a 12-week qigong intervention, the experimental group participants had significant improvement in thinking operations as measured by LOTCA-G, an assessment tool that assessed neurological deficits and mental health problems of our participants. Thinking operations refer to the ability of participants to categorize and perform sequencing of pictures. Based on our observation, the improvement in thinking operations is likely to be due to the qigong learning process. Some qigong routines required the participants to translate metaphorical imagery to movements. Frail elders were required to simulate and formulate movements according to the metaphorical hints from the routine names. For example, routine 5 and routine 9 of Yan Chai Yi Jin Ten-Section Brocade are movements that resemble bird and dragon. The learning and practice process might have trained frail elders' thinking operations. Improvement in categorization can help frail elders simplify and structure perception process and thus enhance their ability to deal with the complex and demanding social environment [33]. Sequencing ability is one of the fundamental abilities for instrumental activities of daily living (ADL) such as managing home and medications [34]. This is in line with the result that the participants had perceived improvement in their ADL. However, further studies using more objective assessment of ADL have to be conducted in order to explore if this qigong protocol improves instrumental activities of daily living of elders. Consistent with previous research efforts [31, 32], other dimensions in cognitive functioning such as memory and attention did not show significant changes after qigong practice. The present findings provided preliminary support to the hypothesis that practicing health qigong could bring positive benefits in specific aspect of cognitive functioning in frail elders. More research needed to be done in this area to investigate the mechanism behind such an effect. 

In psychological aspect, the results are similar to our earlier studies [17, 23]. Participants perceived significant improvement in physical health, ADL, and overall health status as measured by the Perceived Benefits Questionnaire after the 12-week qigong practice. Given that most participants (i.e., 86%) did not show clinical depressive symptoms (GDS < 8) at the beginning of our study, it makes sense that no significant impact of health qigong exercise on the level of depression was found in this study. However, a decreasing trend of depression levels was still observed among experimental group participants from mid-assessment to postassessment, whereas an increasing trend was observed in the comparison group participants. This aligns well with our previous findings that qigong could relieve depressive symptoms [23, 24]. 

Significant reduction in RHR and a trend of improvement in right handgrip strength were found in physical and physiological aspects in the present study. Reduction in RHR was consistently found to be significantly reduced after qigong training [10]. RHR is associated with cardiovascular disease mortality [35]. Its drop will lower one's risk in having all-cause and cardiovascular disease mortality. This suggests that practice of this qigong protocol may have positive effects on heart health of the participants. Further studies using more sophisticated cardiovascular measurements should be conducted to explore this potential therapeutic effect. The trend of improvement in right handgrip was consistent with our earlier RCTs [24]. Less obvious physical and physiological effects observed in the present study might be due to the less physically demanding nature of the current qigong protocol tailor-made for frail elders. 

There are a number of limitations that deserve our attention. First, our actual number of participants in each group did not reach the figure suggested by power analysis. This may be the reasons why some outcome measures were found to be not significant. Second, gender bias constituted a major limitation of the current study. Given that the elderly residential care center and day care center were female dominant, we were unable to recruit male participants during the field test. Nonetheless, no adverse effects were reported during the RCT study. Third, the therapeutic effects in psychological, cognitive, physical, and physiological domains were not maintained in the follow-up period, which is in line with our previous findings [23]. This might be due to a lack of practice after the intervention period. Unfortunately, we did not have records on participants' practice pattern that has limited us to further understand the relationship between continuing practice and maintenance effects of qigong exercise.

## 5. Conclusions

It may be concluded that the new qigong protocol is safe and easy to learn among frail elders. It brings health benefits to frail elders in cognitive, psychosocial, physical, and physiological aspects. Finally, it may be advocated as an activity therapy program to help prevent health-related deterioration in clinical, residential, and community settings. 

## Figures and Tables

**Table 1:** Yan Chai Yi Jin Ten Section Brocades protocol.

New protocol content	Established qigong protocol reference
Routine 1: Wei Tuo presents a Club 1 *韋馱獻杵第一勢*	Yi Jin Jing *易筋經*
Routine 2: Wei Tuo presents a Club 2 *韋馱獻杵第二勢*	Yi Jin Jing *易筋經*
Routine 3: prop up the sky with both hands to regulate the triple warmer *兩手托天理三焦*	Eight-Section Brocades *八段錦*
Routine 4: look back to treat five strains and seven impairments *五勞七傷往後瞧*	Eight-Section Brocades *八段錦*
Routine 5: show claws and flash wings *出爪亮翅*	Yi Jin Jing *易筋經*
Routine 6: pull toes with both hands to reinforce kidney and waist *兩手攀足固腎腰*	Eight-Section Brocades *八段錦*
Routine 7: three plates drop to the ground *三盤落地*	Yi Jin Jing *易筋經*
Routine 8: Clench fists and look with eyes wide open to build up strength and stamina *攢拳怒目增氣力*	Eight-Section Brocades *八段錦*
Routine 9: green dragon extends claws *青龍探爪*	Yi Jin Jing *易筋經*
Routine 10: rise and fall on tiptoes to dispel all diseases *背後七顛百病消*	Eight-Section Brocades *八段錦*

**Table 2:** Content validation ratios (CVRs) for potential therapeutic value of the Yan Chai Yi Jing Ten Section Brocades among six experts.

On therapeutic values: the Yan Chai Yi Jing Ten-Section Brocades protocol	CVR	
	Standing	Sitting
*Psychosocial *		
Facilitates relaxation	1.00*	1.00*
Relieves unpleasant feeling	1.00*	1.00*
Develops confidence to deal with disabilities and medical conditions	0.50	0.67
Culturally relevant activity and arouses interest	1.00*	1.00*
Promotes social contact	0.83*	0.83*
*Physical and physiological aspects *		
Enforces “deep and slow” breathing	1.00*	1.00*
Enforces coordination between respiration and movements	1.00*	1.00*
Enforces trunk, neck and upper limbs stretch	0.83*	0.83*
Promotes functional mobility and balance	1.00*	0.67
Movements can be easily picked up by elders	0.50	0.50
Easy to learn as routine exercise	0.83*	0.83*
*General health *		
From TCM perspective, it promotes good health through practice of reciprocal movements	1.00*	1.00*
From TCM perspective, it stimulates acupressure points and enforces the flow of “Qi”	0.67	0.67
Promotes physical and psychological health	1.00*	1.00*
*Safety *		
Can be adapted to activity tolerance of each individual	0.83*	0.83*
Can be practiced in old age home	0.83*	0.83*
Adequate precautions to prevent potential harm	1.00*	1.00*

*Indicating statements with consensus agreement among the experts, that is, CVR within 0.75–1.00.

**Table 3:** Demographic characteristics of experimental and comparison groups.

Demographic (score range)	Mean (SD) or count (%)		*t* or *χ* ^2^	df	*P* value
	Experimental (*N* = 61)	Control (*N* = 55)			
Age	83.33 (6.30)	84.85 (6.03)	*t* = −1.31	112	0.19
MMSE score	23.69 (3.52)	23.58 (3.61)	*t* = 0.16	114	0.87
CAPE score	9.21 (2.08)	9 (1.98)	*t* = 0.56	114	0.57
GDS score	4.07 (3.11)	4.36 (3.26)	*t* = −0.50	114	0.61
Frailty index	17.75 (6.03)	19.85 (5.43)	*t* = −1.96	114	0.052
Center			*χ* ^2^ = 0.25	1	0.61
Residential care homes	51 (83.6%)	44 (80%)			
Day care center	10 (16.4%)	11 (20%)			
Gender			*χ* ^2^ = −0.50	1	0.61
Male	14 (23%)	15 (27.3%)			
Female	47 (77%)	40 (72.7%)			
Marital status			*χ* ^2^ = 0.85	2	0.66
Single	3 (4.9%)	5 (9.1%)			
Widowed	42 (68.9%)	35 (63.6%)			
Married, spouse alive	16 (26.2%)	14 (25.5%)			
Education level			*χ* ^2^ = 1.28	2	0.53
No school completed	15 (24.6%)	17 (30.9%)			
Primary	38 (62.3%)	28 (50.9%)			
Secondary	8 (13.1%)	9 (16.4%)			
Walking ability			*χ* ^2^ = 0.87	4	0.93
Walk independently	10 (16.4%)	10 (18.2%)			
Walk with stick	19 (31.1%)	15 (27.3%)			
Walk with frame	7 (11.5%)	4 (7.3%)			
Walk with rollator	7 (11.5%)	7 (12.7%)			
Wheelchair bounded	18 (29.5%)	18 (32.7%)			

Notes: MMSE: Mini-Mental State Examination; CAPE: Clifton Assessment Procedures for the Elderly; GDS: Geriatric Depression Scale.

**Table 4:** Acute intervention effects on psychosocial measures.

Outcome	Means and SDs (in brackets)							Repeated measures ANOVA				
(Score range)	Pre-Ax		Mid-Ax		Post-Ax			(Group by time interaction)		(Time interaction)		
	Exp	Com	Exp	Com	Exp	Com	*F *	df	*P*-value		*η*_*p*_^2^	Power
GDS (0–15)	4.07	4.36	4.61	4.24	4.31	4.76	1.16	2,228	0.32	—	0.01	0.25
(Exp: *n* = 61; Com: *n* = 55)	(3.11)	(3.26)	(2.91)	(3.23)	(3.33)	(3.52)						
*Perceived benefits *												
Physical health	—	—	17.09	—	18.05	—	13.01	1,54	—	0.001**	0.19	0.94
(Exp: *n* = 55)			(1.97)		(2.37)							
Activities of daily living	—	—	12.98	—	13.48	—	5.32	1,43	—	0.026*	0.11	0.62
(Exp: *n* = 44)			(1.58)		(1.69)							
Psychological	—	—	25.22	—	25.36	—	0.05	1,57	—	0.82	0.00	0.06
(Exp: *n* = 58)			(4.49)		(3.08)							
Social relationship	—	—	9.84	—	9.91	—	0.32	1,57	—	0.58	0.01	0.09
(Exp: *n* = 58)			(1.09)		(1.05)							
Overall health status	—	—	6.95	—	7.43	—	15.26	1,57	—	0.0001***	0.21	0.97
(Exp: *n* = 58)			(1.08)		(1.22)							

Notes: Com: comparison group; Exp: experimental group; *P < 0.05; **P < 0.01; ***P < 0.001. GDS: Geriatric Depression Scale; only experimental group is required to complete the 21-item Perceived Benefit Questionnaire.

**Table 5:** Acute intervention effects on cognitive measures.

Outcome	Means and SDs (in brackets)						Repeated measures ANOVA (group by time interaction)				
(Score range)	Pre-Ax		Mid-Ax		Post-Ax						
	Exp	Com	Exp	Com	Exp	Com	*F *	df	*P*-value	*η*_*p*_^2^	Power
LOTCA-G											
(Exp: *n* = 61; Com: *n* = 55)											
Orientation (0–16)	14.13	13.87	14.31	13.91	14.11	13.38	0.94	1.9,216.10	0.39	0.01	0.21
	(1.98)	(2.10)	(2.15)	(1.86)	(1.73)	(2.19)					
Perception (0–28)	25.87	25.76	25.67	25.49	26.20	25.91	0.13	2,228	0.88	0.00	0.07
	(2.00)	(2.34)	(2.07)	(2.40)	(2.00)	(2.44)					
Praxis (0–12)	9.57	9.75	9.23	9.35	9.54	9.64	0.04	2,228	0.96	0.00	0.06
	(1.28)	(1.06)	(1.04)	(1.27)	(1.15)	(1.32)					
Visuomotor organization (0–24)	16.75	17.45	16.59	16.87	16.90	16.78	1.58	2,228	0.21	0.01	0.33
	(3.58)	(4.03)	(3.34)	(3.62)	(3.65)	(4.35)					
Thinking operations (0–8)	4.41	4.56	4.49	4.22	4.79	4.22	4.05	2,228	0.02*	0.03	0.72
	(1.80)	(1.69)	(1.84)	(1.64)	(1.83)	(1.70)					
Memory (12)	10.54	10.15	10.52	10.55	10.89	10.84	0.96	2,228	0.39	0.01	0.22
	(1.48)	(1.88)	(1.97)	(2.18)	(1.83)	(1.57)					
Attention (4)	3.84	3.76	3.77	3.87	3.74	3.73	1.26	2,228	0.29	0.01	0.27
	(0.45)	(0.47)	(0.59)	(0.43)	(0.48)	(0.49)					

Notes: Com: comparison group; Exp: experimental group; *P < 0.05.

LOTCA-G: Lowenstein Occupational Therapy Cognitive Assessment-Geriatric.

**Table 6:** Acute intervention effects on physical and physiological measures.

Outcome	Means and SDs (in brackets)						Repeated measures ANOVA (group by time interaction)				
(Score range)	Pre-Ax		Mid-Ax		Post-Ax						
	Exp	Com	Exp	Com	Exp	Com	*F *	df	*P*-value	*η*_*p*_^2^	Power
Right handgrip strength	16.79	15.37	16.50	14.63	17.03	15.00	0.51	1.72,188.93	0.58	0.01	0.13
(Exp: *n* = 58; Com: *n* = 54)	(6.24)	(5.70)	(6.59)	(4.93)	(6.74)	(5.18)					
Left handgrip strength	13.70	13.90	14.02	13.63	14.24	14.24	0.36	2,206	0.70	0.004	0.11
(Exp: *n* = 54; Com: *n* = 51)	(5.57)	(5.46)	(5.23)	(4.74)	(5.76)	(4.64)					
Timed Up & Go	23.28	21.91	22.33	21.55	22.18	22.17	0.54	1.59,119.13	0.54	0.01	0.13
(Exp: *n* = 42; Com: *n* = 35)	(12.75)	(13.05)	(11.91)	(12.12)	(12.71)	(11.72)					
Systolic blood pressure	143.78	148.42	138.03	147.28	136.20	139.78	1.54	2,228	0.22	0.01	0.33
(Exp: *n* = 61; Com: *n* = 55)	(22.20)	(18.45)	(20.04)	(18.74)	(20.94)	(16.72)					
Diastolic blood pressure	76.55	80.13	74.03	77.62	72.82	75.52	0.12	1.89,215.53	0.88	0.00	0.07
(Exp: *n* = 61; Com: *n* = 55)	(13.59)	(12.54)	(10.68)	(11.11)	(11.21)	(10.11)					
Resting heart rate	76.39	75.07	75.47	73.95	73.51	75.04	3.14	2,228	0.045*	0.03	0.60
(Exp: *n* = 61; Com: *n* = 55)	(14.80)	(9.47)	(14.45)	(9.26)	(13.00)	(10.93)					
Forced vital capacity (max)	1.80	1.40	—	—	1.64	1.38	0.79	1,54	0.38	0.01	0.14
(Exp: *n* = 27; Com: *n* = 26)	(0.62)	(0.45)			(0.62)	(0.47)					
Forced expiratory volume in 1s	1.12	0.91	—	—	1.10	1.04	2.75	1,54	0.10	0.05	0.37
(Exp: *n* = 27; Com: *n* = 26)	(0.38)	(0.36)			(0.39)	(0.37)					
Forced expiratory volume in 1s %	64.61	65.93	—	—	70.40	76.00	0.86	1,54	0.36	0.02	0.15
(Exp: *n* = 27; Com: *n* = 26)	(19.34)	(19.39)			(18.16)	(12.44)					

Notes: Com: comparison group; Exp: experimental group; *P < 0.05.
